# Spatial mutual nearest neighbors for spatial transcriptomics data

**DOI:** 10.1093/bioinformatics/btaf403

**Published:** 2025-07-26

**Authors:** Haowen Zhou, Pratibha Panwar, Boyi Guo, Caleb Hallinan, Shila Ghazanfar, Stephanie C Hicks

**Affiliations:** Bioinformatics and Systems Biology Graduate Program, University of California San Diego, Gilman, CA 92093, United States; School of Mathematics and Statistics, The University of Sydney, Camperdown, NSW 2006, Australia; Sydney Precision Data Science Centre, The University of Sydney, Camperdown, NSW 2006, Australia; Charles Perkins Centre, The University of Sydney, Camperdown, NSW 2006, Australia; Department of Biostatistics, Johns Hopkins Bloomberg School of Public Health, MD 21205, United States; Department of Biomedical Engineering, Johns Hopkins University, MD 21218, United States; School of Mathematics and Statistics, The University of Sydney, Camperdown, NSW 2006, Australia; Sydney Precision Data Science Centre, The University of Sydney, Camperdown, NSW 2006, Australia; Charles Perkins Centre, The University of Sydney, Camperdown, NSW 2006, Australia; Department of Biostatistics, Johns Hopkins Bloomberg School of Public Health, MD 21205, United States; Department of Biomedical Engineering, Johns Hopkins University, MD 21218, United States; Center for Computational Biology, Johns Hopkins University, MD 21218, United States; Malone Center for Engineering in Healthcare, Johns Hopkins University, MD 21218, United States

## Abstract

**Motivation:**

Mutual nearest neighbors (MNN) is a widely used computational tool to perform batch correction for single-cell RNA-sequencing data. However, in applications such as spatial transcriptomics, it fails to take into account the 2D spatial information.

**Results:**

Here, we present *spatialMNN*, an algorithm that integrates multiple spatial transcriptomic samples and identifies spatial domains. Our approach begins by building a *k*-nearest neighbors (kNN) graph based on the spatial coordinates, prunes noisy edges, and identifies niches to act as anchor points for each sample. Next, we construct a MNN graph across the samples to identify similar niches. Finally, the spatialMNN graph can be partitioned using existing algorithms, such as the Louvain algorithm to predict spatial domains across the tissue samples. We demonstrate the performance of spatialMNN using large datasets, including one with *N *= 31 10x Genomics Visium samples. We also evaluate the computing performance of spatialMNN to other popular spatial clustering methods.

**Availability and implementation:**

Our software package is available on GitHub (https://github.com/Pixel-Dream/spatialMNN). The code is available on Zenodo (https://doi.org/10.5281/zenodo.15073963).

## 1 Introduction

Recent advances in spatially resolved transcriptomics (SRT) have transformed the landscape for how we measure gene expression in intact tissue (Rao *et al.* 2021, Moses and Pachter 2022). These technologies can profile gene expression at a sub-, near-, or cellular resolution depending on the spatial technology used (Asp *et al.* 2020, Atta and Fan 2021). Unsupervised clustering has become a standard data analytic step in the analysis of SRT data, where the aim is to partition spatial units, cells for image-based technologies, or spots for sequencing-based technologies, measured in the 2D space into either cell types or spatial domains. These cell types or spatial domains can be further explored in downstream analyses, like using differential expression across spatial domains to identify key marker genes. As SRT technologies are used to profile tens to hundreds of tissue sections from different samples, an important challenge is to integrate multiple SRT samples across technical batches and simultaneously identify consistent spatial domains in population- or atlas-scale datasets (Guo *et al.* 2025).

Currently, there are two categories of existing computational methods to integrate multiple SRT samples: (i) methods that were developed to integrate multiple single-cell RNA-sequencing (scRNA-seq) samples across batches (Blondel *et al.* 2008, Korsunsky *et al.* 2018, Haghverdi *et al.* 2018, Welch *et al.* 2019, Luecken *et al.* 2022), and (ii) more modern methods that were developed for the analysis of SRT data by taking into account additional features beyond gene expression counts, including spatial coordinates or image features (Zhao *et al.* 2021, Hu *et al.* 2021, Li and Zhou, 2022, Liu *et al.* 2023, Singhal *et al.* 2024, Xia *et al.* 2023, Yuan 2024). While the first category of robust methods is widely used in practice for the analysis of scRNA-seq data, they fail to leverage the spatial information, which has been shown to be important for downstream analyses (Li *et al.* 2021, Yuan *et al.* 2024, Hu *et al.* 2024). In addition, these more modern tools can be technology-dependent and computationally inefficient, including both running time and memory usage (Yuan *et al.* 2024, Hu *et al.* 2024). Finally, existing benchmark evaluations to spatially cluster SRT samples have primarily evaluated performance within a tissue section or utilized a method such as PASTE (Zeira *et al.* 2022) to physically align two tissue sections before performing spatial clustering.

To address these problems, we developed spatialMNN, an algorithm that integrates multiple SRT samples using graph-based approaches and then identifies spatial domains across the SRT samples jointly. Our approach leverages mutual nearest neighbors (MNN) (Haghverdi *et al.* 2018), which is used by many computational tools (Korsunsky *et al.* 2018, Butler *et al.* 2018, Lin *et al.* 2019) and has consistently been recognized for its high performance in both accuracy and scalability in terms of memory efficiency and speed (Luecken *et al.* 2022). We demonstrate the performance of spatialMNN using both simulated and real SRT datasets, which scales to large-scale SRT datasets. We also evaluate the computing performance of spatialMNN compared to other popular spatial clustering methods.

## 2 Methods

### 2.1 Summary of the spatialMNN algorithm

#### 2.1.1 Preprocessing, dimensionality reduction, and spatial graph construction

**Figure 1.**
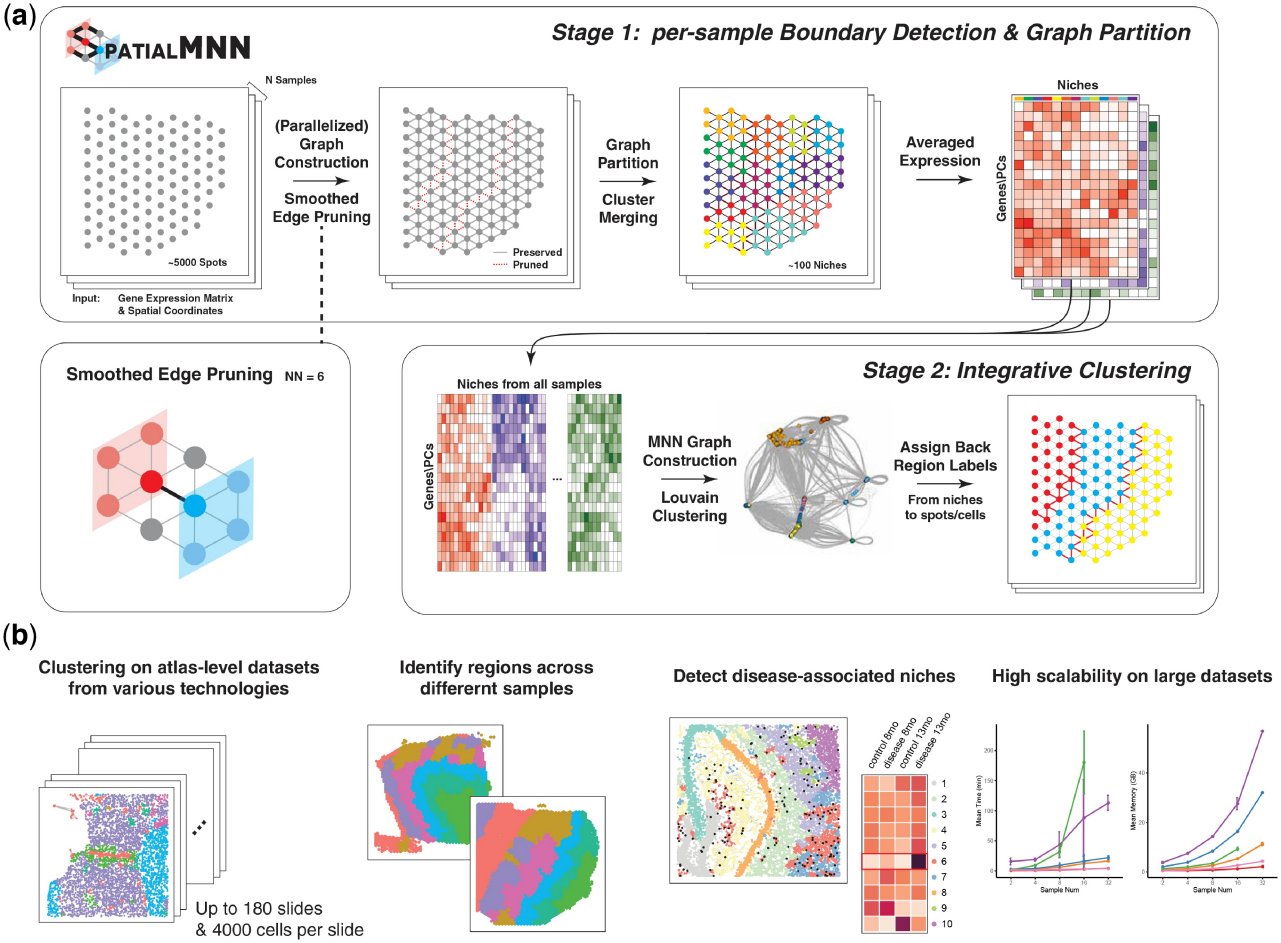
Overview of spatialMNN to integrate multiple SRT samples and perform downstream analyses including spatial domain detection. (a) Given a set of *N* multi-sample SRT datasets, the spatialMNN algorithm builds a *k*-nearest neighbor (kNN) graph based on the spatial coordinates and gene expression within each tissue sample. Next, edge weights are smoothed (considering neighboring spots/cells) and pruned to identify a set of *anchor points* for each sample. Then, spatialMNN constructs a MNN graph across the samples, followed by Louvain clustering to identify similar niches across the samples using gene expression that has been averaged across spots/cells within a niche. The resulting clusters are assigned back to the original spatial coordinates. (b) spatialMNN can be used in downstream analyses, including detecting spatial domains in large-scale atlas datasets, identifying regions across different samples, and detecting disease-associated niches. We demonstrate how spatialMNN is highly scalable and accurate on large datasets.

The spatialMNN workflow begins with preprocessing steps (Fig. 1, available as supplementary data at *Bioinformatics* online). There are two options for these steps. In one option, for each tissue sample, spatialMNN uses GLM-PCA (Townes *et al.* 2019) to perform dimensionality reduction. We use this approach because GLM-PCA has been shown to avoid distortions and potential false discoveries associated with normalization and ${\;\text{log}\;}_{2}$-transformed data that is highly sparse (Townes *et al.* 2019). Specifically, we use the nullResiduals() function from the *scry* R package (F. William Townes, Kelly Street, 2024) to obtain a set of deviance residuals, which are used as input to PCA to calculate a set of top PCs with a default of 30 PCs. Alternatively, a user has the option to identify highly variable genes and use the standard PCA approach to perform dimensionality reduction, if desired. Either way, the output from the dimensionality reduction step is a new matrix $Y^{M\times N}$ containing *M* spots/cells and *N* PCs.

**Figure 2.**
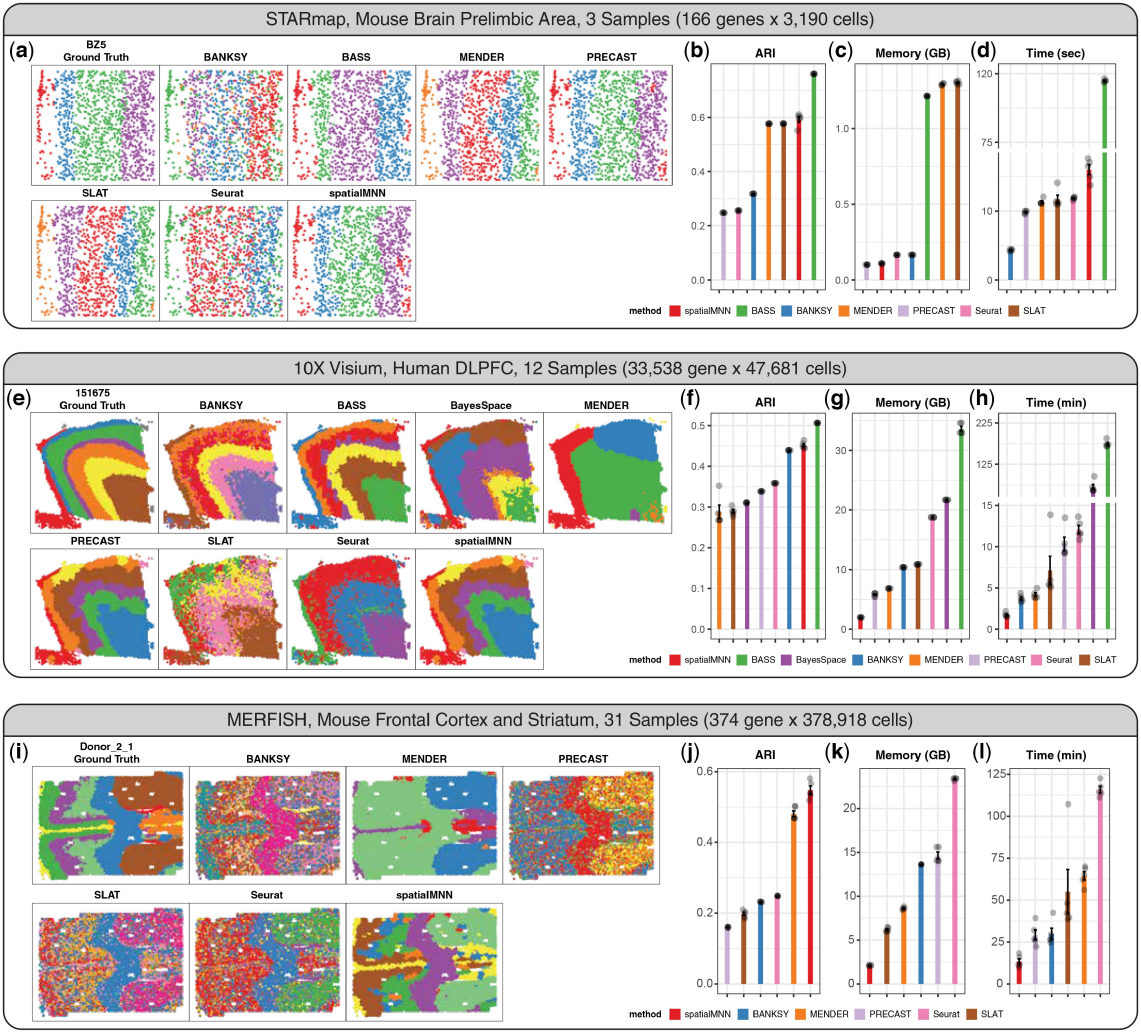
Application of spatialMNN to integrate multiple tissue sections and detect cell types and spatial domains across samples within three datasets measured on the STARmap, Visium, and MERFISH platforms. We considered three datasets (a–d) *n *= 3190 cells across *N *= 3 tissue sections measured on the STARmap platform (Wang *et al.* 2018), (e–h) *n *= 47 681 spots across *N *= 12 tissue sections measured on the 10× Genomics Visium platform (Maynard *et al.* 2021), and (i–l) *n *= 378 918 cells across *N *= 31 tissue sections measured on the MERFISH platform (Joung *et al.* 2023). (a, e, i) Visualization of one tissue section from each dataset colored by the manually labeled (‘Ground Truth’) or predicted cell types/domains from spatialMNN, BASS (Li and Zhou 2022), PRECAST (Liu *et al.* 2023), BayesSpace (Zhao *et al.* 2021), BANKSY (Singhal *et al.* 2024), SLAT (Xia *et al.* 2023), MENDER (Yuan, 2024), and Louvain (Blondel *et al.* 2008) in Seurat. Performance evaluation (*y*-axis) of (b, f, j) adjusted Rand index (ARI) comparing the performance of the algorithms (*x*-axis) to the manual annotations (repeated 5 times), (c, g, k) elapsed time (minutes) for each of the algorithms (*x*-axis), (d, h, l) maximum memory (RAM) used (GB) for each of the algorithms (*x*-axis). Louvain (Seurat) and BASS are excluded in the MERFISH frontal cortex benchmark due to out-of-memory issue. BayesSpace is excluded in STARmap brain prelimbic area and MERFISH frontal cortex benchmarks due to application limitation. All benchmarks are run on a high performance computing cluster (8 cores, 128-GB max memory, and 36-h max running time).

Next, using the euclidean distance of the spatial coordinates of spots/cells, the spatialMNN algorithm constructs a *k*-nearest neighbors (kNN) graph (referred to as a ‘spatial graph’) using the kNN() function from the *dbscan* R package (Hahsler *et al.* 2019, Hahsler and Piekenbrock 2024). The parameter *k* is flexible (default k=6) and can be decided from SRT platforms or cell density (Fig. 2, available as supplementary data at *Bioinformatics* online). We denote the kNN graph as G=<V,E>, where *V* represents the set of all spots/cells and *E* the set of edges connecting each node u∈V with its *k*-nearest neighbors. The edge weights are calculated based on Pearson’s correlation scores between the PCs of spot pairs (default), which works well for most types of datasets. Alternatively, the number of SNN in the PC space is useful for more sparse datasets, such as cases where more genes have zero expression (zero-inflated), or where the weight calculations based on Pearson’s coefficient may fail.

#### 2.1.2 Smoothed edge pruning

Next, spatialMNN prunes (or removes) edges either (i) using a fixed threshold (removing edges below 0.6 as a default) or (ii) alternatively, users can use a smoothed edge pruning algorithm. A graphical summary of the algorithm is provided in Fig. 2, available as supplementary data at *Bioinformatics* online and detailed below, but the motivation of the smoothed version of edge pruning is to minimize the effects of noisy expression at the spot/cell level by considering adjacent edges of a specific edge *e* when calculating the *e*th edge weight.

Our smoothed edge pruning algorithm aims to identify adjacent point sets for each edge by assigning edge weights and determining whether an edge should be pruned, while also identifying biologically significant boundaries. Specifically, for a given edge *e*, the connecting nodes (u,v), and the set of (u,v)’s *q* nearest neighbors in the spatial domain denoted as $V_{u}$ and $V_{v}$, the edge weight is computed as follows:

$$W_{e}=\mathrm{Corr}(\mathrm{Avg}(Y_{\left\{V_{u}-V_{u}\cap V_{v},u\right\}}),\mathrm{Avg}(Y_{\left\{V_{v}-V_{u}\cap V_{v},v\right\}})),$$

where $Y^{M\times N}$ is a matrix containing *M* spots/cells and *N* PCs.

**Figure 3.**
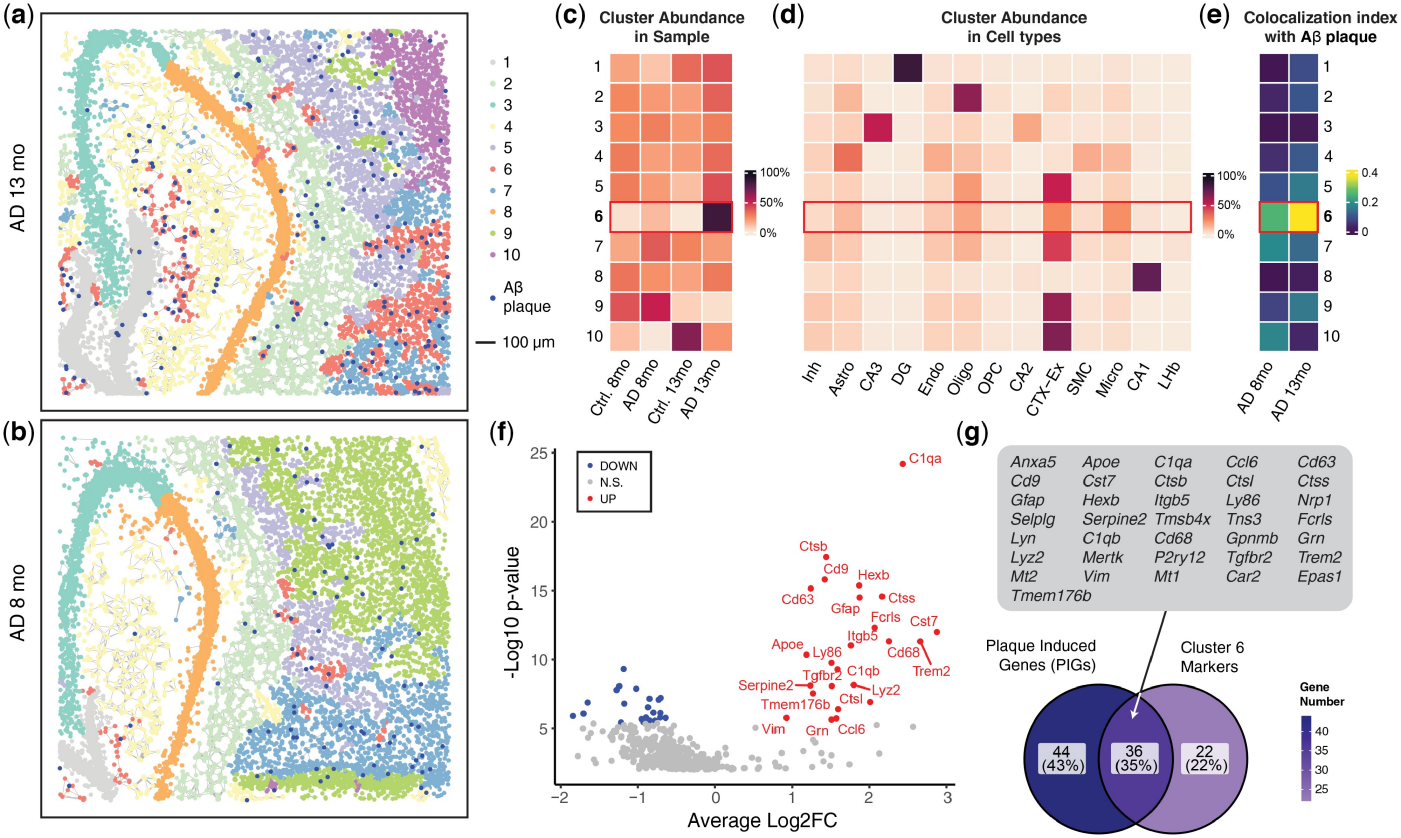
SpatialMNN identifies disease-related niches in mouse AD samples. STARmap PLUS was used to profile the gene expression and protein (in the same tissue) from *N *= 4 mouse samples (Control (*N *= 2) and Alzheimer’s disease (AD) (*N *= 2) mice at ages 8 months (mo) and 13 mo) (Zeng *et al.* 2023). (a, b) To integrate and perform multi-sample spatial clustering, spatialMNN identified 10 predicted niches (or unsupervised clusters) in two AD samples at (a) 13 months and (b) 8 months, with dark blue spots indicating Aβ plaques (protein). The plotting scale is shown in the legend. (c) Heatmap of the cluster abundance across samples, highlighting the increased presence of cluster 6 in AD samples (compared to control samples), especially in the 13-mo sample, suggesting cluster 6 is a disease-associated niche. (d) Heatmap of the cluster abundance across cell types, suggesting the microglia (Micro), astrocytes (Astro), and oligodendrocytes (Oligo) cell types are enriched in cluster 6. (e) Co-localization index heatmap shows the spatial correlation of clusters with Aβ plaques, with cluster 6 having the highest co-localization index. (f) Differential expression (DE) analysis identifies marker genes in cluster 6. (g) Venn diagram comparing the marker genes found in cluster 6 with the Plaque-Induced Genes (PIGs) identified in the original study, showing a significant overlap. All overlap genes were annotated in the gray box.

Next, the edges with weight below the user-defined threshold (default 0.6, can be determined from the distribution of edge weights) (Fig. 3, available as supplementary data at *Bioinformatics* online) are pruned to form the final graph for partitioning in the next step.

#### 2.1.3 Partitioning the spatial graph as a form of data reduction

Following edge pruning, the spatial graph undergoes partitioning via the Louvain algorithm (Blondel *et al.* 2008), set to a high resolution (∼10), resulting in 100–200 small niches for each tissue sample. These niches act as a set of anchor points for the sample, but also shrink the data from potentially thousands of spots/cells to hundreds of small niches. Niches with too few spots/cells (default n<5) will be merged with nearby niches that most of the neighbors belong to. Using these niches, we further reduce the raw gene expression counts matrix by pseudobulking counts across all spots/cell within a given niche/cluster.

#### 2.1.4 Mutual nearest neighbors on the spatial niches

Next, we construct a MNN (Haghverdi *et al.* 2018) graph across the niches identified within each tissue sample. Using the pseudobulked (across spots within a niche) gene expression data, the spatialMNN algorithm will first find each niche’s *k*-nearest neighbors in every sample except itself (or include itself if the user wants to detect sample-specific cell types). Suppose there are a pair of niches from different samples, *u* from sample 1 and *v* from sample 2. The algorithm will find the set, $V_{u}$, which includes *u’*s k-nearest neighbors in sample 2 in PCA space and, $V_{v}$ in the same way. Only if $u\in V_{v}$ and $v\in V_{u}$, will this pair of niches be connected in the MNN graph.

In general, the above approach applies to cells or tissue types that are shared by at least two samples, but if there is a sample-specific cell type, these nodes will not be added to the MNN network and cannot be correctly grouped together, as there are no MNN in other samples. Therefore, it is possible to consider finding mutual neighbors within the sample, so that the niches contain a unique cell type of the sample that can be successfully identified and grouped. After constructing the MNN graph, the graph will also be partitioned using Louvain clustering, but with a lower resolution (∼1). Finally, the clustering label will be assigned to all spots within a labeled niche.

#### 2.1.5 Parallelizing the spatialMNN algorithm

The parallel computing capability of spatialMNN leverages the R *parallel* package to enhance performance. Due to spatialMNN’s two-stage design (Fig. 1, available as supplementary data at *Bioinformatics* online), parallelization is straightforward. In the first stage, since the preprocessing and spatial graph construction steps for each sample are independent, different samples can be assigned to separate cores for processing. Each sample will be copied only once. For datasets with large sample sizes, this approach significantly reduces processing time while keeping memory consumption at a minimal level. In the most extreme case, where the number of available cores matches or exceeds the number of samples, using the parallel version of spatialMNN requires only double the memory for the first stage. To enable parallelization, simply set the core_num parameter to an integer greater than one in the stage_1 function.

#### 2.1.6 Resolve batch effects in large samples

In the second stage of spatialMNN, batch effects are addressed during dimensionality reduction and clustering steps. By using the Pearson residuals approximation to GLM-PCA, spatialMNN partially mitigates batch effects in the data (Townes *et al.* 2019). Additionally, the use of MNN in the clustering process further reduces the impact of batch effects. By default, the MNN construction process only connects similar nodes between different samples, preventing over-connection of nodes within the same sample (Haghverdi *et al.* 2018), which could otherwise result from batch effects. However, spatialMNN includes an option that allows users to enable connections within the same sample if necessary. This option is particularly useful when dealing with small sample sizes or when certain samples contain unique cell or tissue types, thereby improving clustering quality.

### 2.2 Datasets

SpatialMNN can be applied to either (i) image-based, targeted, in situ transcriptomic profiling at a molecular and single-cell resolution or (ii) non-targeted RNA capture and sequencing approaches (Atta and Fan 2021). For a complete description of the publicly available datasets from the (i) mouse brain STARmap data (Wang *et al.* 2018), (ii) human dorsolateral prefrontal cortex data profiled on Visium (Maynard *et al.* 2021), (iii) MERFISH mouse frontal cortex and striatum data (Joung *et al.* 2023), (iv) STARmap PLUS AD mouse brain data (Zeng *et al.* 2023), (v) MERFISH mouse hypothalamic preoptic region data (Moffitt *et al.* 2018), and (vi) human hippocampus data profiled on Visium (Nelson *et al.* 2024), see Note 1, available as supplementary data at *Bioinformatics* online.

#### 2.2.1 Generation, preprocessing, and analysis of simulated data

As the dataset that is manually annotated may still have inaccurate or false annotations, we also considered simulating data to assess the accuracy of each method, because we can define the ground truth for each sample. Further, since we can control the size of the generated sample, we can evaluate the efficiency of the algorithm, including the amount of time and memory that is consumed during the operation. Time and memory consumption are both of utmost importance as they determine how well an algorithm can scale when facing the challenge of larger datasets in the future. In addition, we can artificially introduce batch effects during data simulation to test the robustness of each algorithm for batch effects.

The data generation involves two phases (Fig. 5). The first phase determines the spot array (the position for each spot), the tissues and cell types contained in the generated data, and their spatial distribution. Here, a tissue is defined as a region containing multiple cell types, aligning more closely with real-world scenarios. The spatial distribution patterns of the tissues typically include layered or ringed structures, with the specific size of the distribution area determined manually. A sample usually comprises thousands of units (each unit consisting of coordinates and transcriptomic information). Each tissue can contain multiple cell types. The proportions of these cell types can be manually set and represented by constructing a probability matrix of tissue-cell type, as shown in Table 1.

**Table 1.** The proportion of cell types for each simulated group/spatial domain (described in Section 4.2.1 and visualized in Fig. 5b).

	Cell type 1	Cell type 2	Cell type 3	Cell type 4
Group 1	0.90	0.03	0.04	0.03
Group 2	0.20	0.60	0.05	0.15
Group 3	0.25	0.15	0.5	0.10
Group 4	0.10	0.10	0.10	0.70

The second phase involves generating SRT data based on the tissue-cell type probability matrix, that is, determining the cell type or composition for the units generated in the previous phase. To reflect the differences in data generated by different technologies, in a cell-based dataset, a unit contains only one cell. In contrast, in a spot-based dataset, a unit consists of several cells, represented by averaged expression profile data. The cell types of these cells are randomly generated based on the aforementioned probability matrix. For transcriptomic data, each cell type has 200 highly expressed genes (referred to as marker genes). There are also 10% noisy genes added to simulate real-world scenarios. Regarding gene expression, the marker genes of a particular cell type will be highly expressed in the corresponding cells, while all other genes maintain a zero expression level. After generation, all expression values will have an added layer of sample-wise shift and Gaussian noise to simulate real-world situations and batch effects. The formula for the data generation process is described below:

$$X_{i,j}=\frac{1}{N_{i}}\sum_{k=1}^{N_{i}}\delta (L_{k},L_{g})\times E_{g}+E_{s}+\epsilon ,\epsilon ∼N(0,\sigma^{2}+\sigma_{s}^{2}),$$

where *i*, *j* stand for the index of the unit and gene(feature), $N_{i}$ is the number of cells in the unit, $L_{k},L_{g}$ are the cell type label of cell *k* and the cell type to which *g’*s marker gene list belongs, and δ is the Kronecker delta function which outputs 1 when the two inputs are equal and 0 otherwise. $E_{s}$ and $\sigma_{s}^{2}$ are sample-specific shift and variance factors to introduce batch effects.

### 2.3 Existing computational methods

We compared spatialMNN to existing computational methods: BASS (Li and Zhou 2022), PRECAST (Liu *et al.* 2023), BayesSpace (Zhao *et al.* 2021), BANKSY (Singhal *et al.* 2024), SLAT (Xia *et al.* 2023), and MENDER (Yuan 2024) that integrate multiple SRT tissue samples and perform unsupervised clustering (see Note 2, available as supplementary data at *Bioinformatics* online). In our benchmark evaluations, we used default parameters obtained from the developer-provided tutorials or publications, unless noted below.

### 2.4 Overview of metrics used for evaluation

#### 2.4.1 Performance metrics

We used the ARI to compare the overlap of the manual annotations at a spot/cell resolution to a predicted cell type or spatial domain. An ARI of 0 corresponds to random labeling, and an ARI of 1 corresponds to perfect agreement. We used the adjustedRandIndex() function in the *mclust* R package (Scrucca *et al.* 2023) (version 6.0.0). We also used NMI to evaluate the accuracy using our simulated SRT samples. An NMI of 0 corresponds to no mutual information and 1 corresponds to perfect correlation. We used the NMI() function in the *aricode* R package (Chiquet *et al.* 2023) (version 1.0.3).

#### 2.4.2 Spatial feature-related metrics

In Fig. 3 (the analysis of AD mouse datasets), we used the co-localization index to demonstrate the spatial relationship of identified domains with Aβ plaque. The index of a certain domain is calculated by (1) counting the nearby plaques (<20 μm) for each cell and (ii) averaging the plaque number by domains.

#### 2.4.3 Computational environment

In R R-related benchmark, we evaluated both the max or peak memory used and computing time using the peakRAM() function in the peakRAM R package (Quinn 2017) and the Sys.time() function in base R (version 4.3/4.4), respectively. We performed all benchmark evaluations on a high-performance computing cluster with 8 cores and a max of 128-GB memory.

### 2.5 Software availability

spatialMNN is freely available as an R package on GitHub (https://github.com/Pixel-Dream/spatialMNN) and will be submitted to Bioconductor. Code to reproduce all preprocessing, analyses, and figures in this manuscript is available from GitHub at https://github.com/Pixel-Dream/spatialMNN_analysis. The complete code and data required to reproduce the analysis are available on Zenodo with the doi: 10.5281/zenodo.15073963. We used spatialMNN version 0.99.0 for the analyses in this manuscript.

## 3 Results

### 3.1 spatialMNN for batch correction and detecting spatial domains

In a similar spirit to MNN (Haghverdi *et al.* 2018), the spatialMNN algorithm is designed to integrate multiple transcriptomics datasets, except spatialMNN is designed for transcriptomics datasets with spatial coordinate information, which can be used in downstream analyses to identify shared spatial domains (or clusters) across the samples (Fig. 1, Fig. 1, available as supplementary data at *Bioinformatics* online). Broadly, our approach begins by building a kNN graph based on the spatial coordinates within each tissue sample. Specifically, we denote the kNN graph as G=<V,E>, where *V* represents the set of all spots/cells and *E* the set of edges connecting each node u∈V with its *k*-nearest neighbors. Using edge weights measured by Pearson’s correlation or the number of Shared Nearest Neighbors (SNN) on gene expression, we prune noisy edges using a novel smoothed edge pruning algorithm (Figs. 2 and 3, available as supplementary data at *Bioinformatics* online), described in greater detail in Section 4.1. Using these pruned edges, spatialMNN identifies niches to act as *anchor points* for each sample. Next, we construct an MNN graph across the samples to identify similar niches across the samples. Finally, the spatialMNN graph can be partitioned using existing algorithms, such as the Louvain algorithm, to predict spatial domains across the tissue samples.

### 3.2 Key innovations of spatialMNN

The key innovations of spatialMNN compared to existing approaches are as follows. First, recent work (Guo *et al.* 2025) described challenges to integrate multiple SRT samples, including how existing approaches that perform multi-sample batch correction for SRT use existing methods developed for scRNA-seq data (Korsunsky *et al.* 2018, Haghverdi *et al.* 2018, Welch *et al.* 2019, Luecken *et al.* 2022). A key innovation of spatialMNN is that it takes into account spatial coordinates as input, in addition to the gene expression, making it uniquely designed for SRT data. Second, due to spatialMNN being a graph-based approach, it can be applied to either non-targeted RNA capture and sequencing, such as Slide-seq or 10× Genomics Visium, or image-based, targeted, *in situ* transcriptomic profiling at a molecular and single-cell resolution, such as MERFISH or Xenium (Atta and Fan, 2021). Third, because a divide-and-conquer approach of first identifying spatial niches within each sample separately is used, the computational complexity of spatialMNN is reduced, and the runtime is fast. Specifically, the partitioning of the graph into anchor points (or niches) serves as a form of data reduction, where the information from potentially thousands of spots/cells is reduced to hundreds of anchor points. Similarly, SpaceFlow-DC (Ren *et al.* 2022, Yuan *et al.* 2024) also uses a divide-and-conquer approach, while other methods are designed for one sample at a time or are infeasible to apply to large datasets. As the cost of generating these data decrease, computational complexity and runtime to integrate SRT data across multiple tissues or individuals will become increasingly important to perform population-level analyses (Guo *et al.* 2025).

### 3.3 spatialMNN performs comparably to existing methods in accuracy and outperforms in scalability

**Figure 4.**
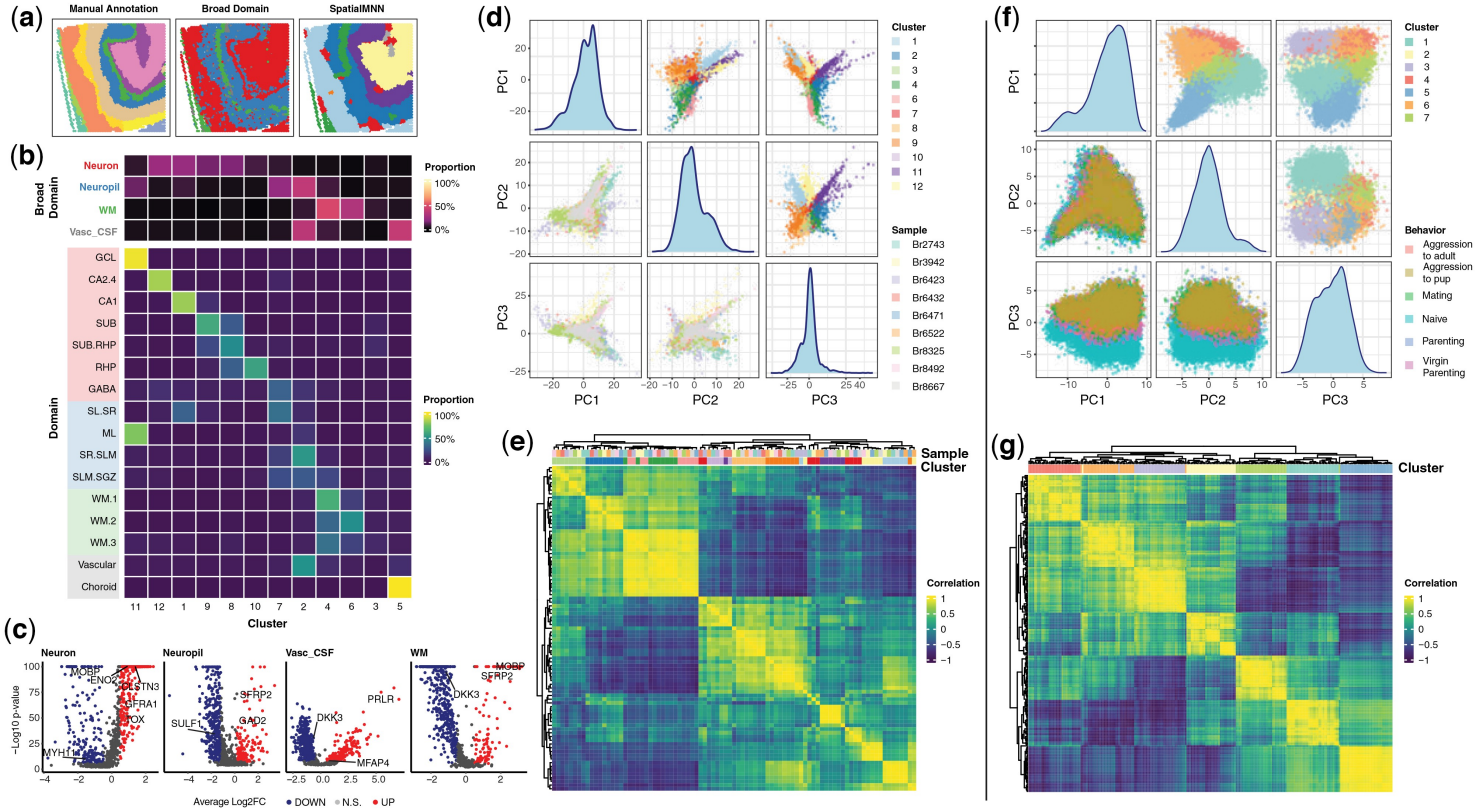
Application of spatialMNN to large SRT datasets with *N *= 31 Visium samples and *N *= 181 MERFISH samples. Panels (a–e) and (f and g) are based on *N *= 31 tissue sections from human hippocampus profiled on the 10× Visium platform (Nelson *et al.* 2024) and *N *= 181 slides (over 870,000 cells) profiled on MERFISH (Moffitt *et al.* 2018), respectively. (a) Spot plots of sample *Br8667_V11L05-336_A1*’s domains using a manual annotation, a data-driven approach to “broad domain”, and spatialMNN. (b) Heatmap showing the fraction of different cell types/broad domain types in each cluster identified by spatialMNN. (c) Volcano plots of four broad domains, highlighting differentially expressed genes across identified clusters overlap with the manually annotated domains: Neuron (clusters 1, 8, 9, 12), Neuropil (clusters 4, 8, 12), White Matter (WM; clusters 3, 4, 6), and Vascular_CSF (clusters 2, 5). (d) Distribution of identified clusters and samples in the top three principal components (PCs). (e) Heatmap of Pearson’s correlation demonstrating the consistency of identified clusters based on gene expression profiles. (f) Distribution of identified clusters and sample-level variables projected along the top three PCs, similar to panel (d), showing the robustness of spatialMNN in a larger dataset. (g) Correlation heatmap of identified clusters.

**Figure 5.**
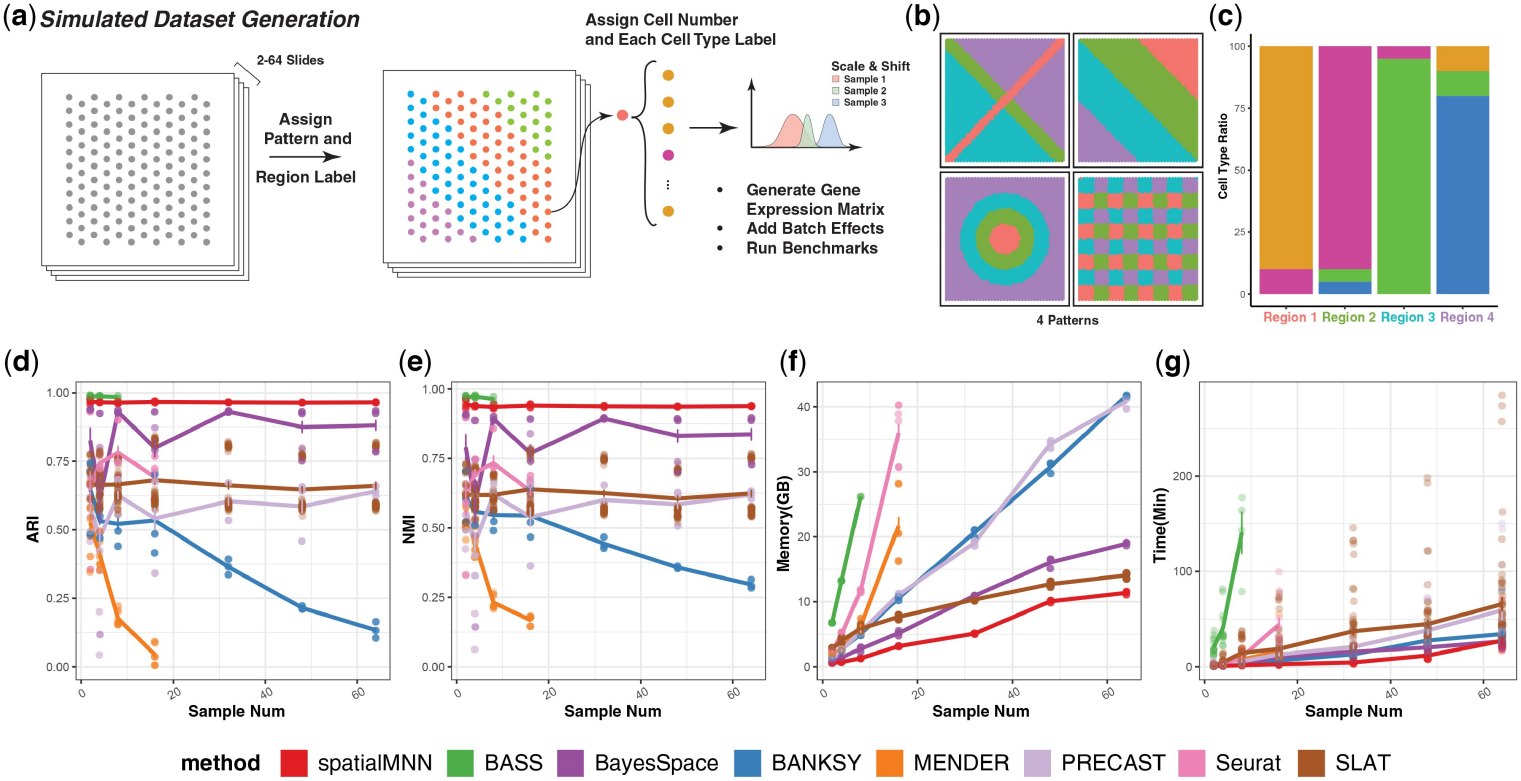
Simulation benchmark reveals the low memory and time efficiency of SpatialMNN. (a) Overview of the simulated dataset generation process: patterns and region labels are assigned to cells across 2–64 slides, with cell numbers and types specified to generate a gene expression matrix with sample-wise batch effects for benchmarking. (b) Illustration of the four distinct spatial patterns used in the simulation. (c) Bar plot showing the cell type composition across four different regions. (d–g) Benchmarking results comparing spatialMNN with other methods (BASS, BANKSY, BayesSpace, MENDER, PRECAST, Seurat, and SLAT) on (d) Adjusted Rand Index (ARI), (e) Normalized Mutual Information (NMI), (f) memory usage, and (g) runtime across different sample sizes. Three series of datasets were simulated with different random seeds. Each simulated benchmark with a different sample size was repeated 5 times. SpatialMNN demonstrates high clustering accuracy and robustness to batch effects with consistently high ARI and NMI scores, while maintaining significantly lower computational costs in terms of runtime and memory usage.

To demonstrate the applicability of spatialMNN on real SRT datasets, we considered three datasets across different technologies and scales. In the first dataset (Wang *et al.* 2018), the authors used the STARmap platform to profile the prelimbic area in the mouse brain across three tissue sections, 166 genes, and 3190 cells (Fig. 2a–d, Fig. 4, available as supplementary data at *Bioinformatics* online). The second dataset (Maynard *et al.* 2021) used the 10x Genomics Visium Spatial Gene expression platform to profile postmortem human dorsolateral prefrontal cortex (DLPFC) across 12 tissue sections, 33 538 genes, and 47 681 spots (Fig. 2e–h, Fig. 5, available as supplementary data at *Bioinformatics* online). In the third dataset (Joung *et al.* 2023), the authors used the MERFISH platform to profile the mouse frontal cortex and striatum across 31 tissue sections, 374 genes, and 378 918 cells (Fig. 2i–l, Fig. 6, available as supplementary data at *Bioinformatics* online). In each of these datasets, the manually labeled cell type or spatial domains (which we use as approximate ground truth) are used to help compare the performance of spatialMNN to existing algorithms (labeled ‘Ground Truth’ in Fig. 2a, e, i). We applied spatialMNN to integrate the multiple tissue sections within each dataset and compared it to existing algorithms that can be used to detect spatial domains across multiple samples, including BASS (Li and Zhou, 2022), PRECAST (Liu *et al.* 2023), BayesSpace (Zhao *et al.* 2021), BANKSY (Singhal *et al.* 2024), SLAT (Xia *et al.* 2023), MENDER (Yuan 2024), and Louvain (Blondel *et al.* 2008). We used the adjusted Rand index (ARI) to evaluate the spatial domains detected by each of the algorithms compared to the manually labeled cell types or spatial domains. We further evaluated the runtime and memory used over the course of the algorithm.

While BASS outperformed spatialMNN in accuracy compared to the manual labels (assessed with ARI) in the first and second dataset (Fig. 2b, f, j), we found the runtime and memory usage requirements for BASS to be significantly higher compared to existing algorithms (Fig. 2c, d, g, h). Specifically, in the first dataset with 3190 cells across 3 tissue sections, BASS took 115 s to complete compared to spatialMNN, which took 16 s. Similarly, in this dataset, BASS required 1243 MB of memory compared to 110 MB with spatialMNN. In the second dataset with 47 681 spots across 12 tissue sections, BASS and spatialMNN used 175 and 1.7 min, along with 33.6 and 1.98 GB, respectively. In the third dataset, we were unable to run BASS entirely due to out-of-memory issue (maximum 128 GB, Fig. 2k and l) while, on average, spatialMNN only used about 2 GB of memory and 15 min to finish the clustering. Finally, when comparing spatialMNN to other existing algorithms, we found that spatialMNN resulted in the highest ARI score along with a quicker running time and smaller memory usage.

### 3.4 spatialMNN identifies disease-associated niches in Alzheimer’s disease

Understanding the specific patterns of the spatial organization of cells can be important to understand the development and the pathology of disease, such as cancer or Alzheimer’s disease (AD). However, existing algorithms for mining these spatial patterns rely on transcriptome-based cell type annotation (Yang *et al.* 2022, Yuan *et al.* 2022). Here, we used spatialMNN in an unsupervised manner to identify disease-associated niches in mouse AD samples (Zeng *et al.* 2023).

Using spatialMNN, we identified unique spatial domains and cell types within mouse brain regions across control and AD tissue (Fig. 3a and b). By comparing the clustering results of AD samples from 13-month (a) and 8-month (b) mice, we observed that cluster 6 emerged as a disease-associated niche, showing a unique spatial distribution pattern related to AD pathology. A heatmap comparing cluster abundance across different samples highlights the increase of cluster 6 in AD samples, especially in the 13-month sample (Fig. 3c). Specifically, we found a progressive enrichment of this disease-associated niche with increasing age and disease severity.

Using published cell type annotations in the original paper (Zeng *et al.* 2023), we found differences in the cell type abundances within the spatialMNN clusters (Fig. 3d). Specifically, we found that cluster 6 is enriched for microglia, astrocytes, and oligodendrocytes. This suggests that these cell types are key components of the identified disease-associated niche, aligning with literature findings (Liddelow *et al.* 2017) and underscoring the qualitative accuracy of spatialMNN in identifying disease-associated niches. Next, we investigated if the predicted clusters were co-localized with the location of the A β plaques using a spatial correlation metric. The spatial co-localization analysis found that cluster 6 resulted in the highest co-localization index with the A β plaques, further emphasizing the relevance of cluster 6 to AD pathology (Fig. 3e).

Finally, we used differential expression (DE) analysis to identify marker genes for cluster 6 (Fig. 3f). We compared top DE genes from this cluster with Plaque-Induced Genes (PIGs), identified in the original study, and found a strong overlap between these gene sets. This suggests consistency between the identified marker genes and previously reported PIGs (Fig. 3g). Overall, we found spatialMNN effectively identified disease-associated niches in the spatial transcriptomics dataset, revealing key spatial and molecular aspects of AD pathology in mouse models.

To assess whether these insights were unique to spatialMNN, we compared its performance with two existing methods: Seurat, widely used in scRNA-seq analysis, and BASS, a high-performing method from our earlier benchmarks. While Seurat was able to identify microglia near plaques, it failed to capture the specific spatial organization pattern associated with AD (Fig. 7a, d, available as supplementary data at *Bioinformatics* online). BASS, by contrast, failed to identify both the relevant cell types and spatial patterns (Fig. 7b, e, available as supplementary data at *Bioinformatics* online). These comparisons highlight spatialMNN’s distinct capability to detect biologically meaningful spatial organization patterns associated with disease, offering insights beyond what existing methods can provide.

### 3.5 spatialMNN in application to atlas-scale datasets

To demonstrate how spatialMNN can scale to datasets with a larger sample size, we used a dataset with *N *= 31 tissue sections from Nelson *et al.* (2024), who used the 10x Genomics Visium Spatial Gene Expression platform to profile postmortem human hippocampus across 10 donors, 31 483 genes, and 150 917 spots. Using spatialMNN, we integrated and identified 13 spatial domains across the *N *= 31 tissue sections. We found the spatialMNN spatial domains were consistent with domains found by the original authors (Fig. 4a and b). Specifically, in the original paper, the authors manually annotated the spots based on known morphology and gene markers along with applying PRECAST (Liu *et al.* 2023) to annotated spots using a data-driven approach to a set of “broad domains.”

Motivated by the use of the defined broad domains in (Nelson *et al.* 2024), we similarly combined the spatial domains detected by spatialMNN to a comparable resolution of “neuron” (clusters 1, 8, 9, 12), “neuropil” (clusters 4, 8, 12), “white matter” (WM; clusters 3, 4, 6), and “vascular_CSF” (clusters 2, 5). We identified DE genes within these broad domains from spatialMNN and found a consistent set of DE genes from the original authors (Fig. 4c). Using the predicted domains from spatialMNN, we performed principal components analysis (PCA) and found that the spatialMNN domains explained the most variation in the top three principal components (PCs), compared to the donor-level variation (Fig. 4d). Furthermore, we found a strong correlation at the gene-expression level across the samples within each cluster (Fig. 4e).

In a second example, we considered a dataset consisting of *N *= 181 slides with over 870 000 cells profiled on the MERFISH platform (Moffitt *et al.* 2018). Similar to the first dataset, we used spatialMNN to detect 7 spatial domains jointly across all 181 slides. We found the spatialMNN domains explained the most amount of variation compared to other known variables (Fig. 4f). Finally, we found a strong correlation at the gene-expression level across the cells from different samples within each cluster (Fig. 4g).

### 3.6 spatialMNN is fast and memory efficient

In addition to evaluating spatialMNN using real SRT samples, we also simulated SRT samples to demonstrate how spatialMNN scales with both running time and memory efficiency. We considered SRT datasets ranging from *N *= 2–64 samples, each with a known set of spatial domains made up as a mixture of cell types within each spatial domain (Fig. 5a). Briefly, we developed a two-stage approach for simulating SRT data (see Section 4.2.1 for more details), starting with four predefined spatial patterns (Fig. 5b) designed to mimic different types of tissue spatial distributions. Next, we simulated the number of cells in each spot using a uniform distribution. Then, we assigned cell types to each cell based on a predefined tissue-to-cell type probability table (Fig. 5c). Each cell type was assigned 200 marker genes, resulting in a total of 800 marker genes. To simulate realistic conditions, we also incorporated 10% noisy genes and batch effects. Finally, for each simulated sample, the resulting gene expression matrix consisted of 889 genes and 4992 cells simulated from Gaussian distributions. We generated a series of seven datasets, with sample sizes of *N *= 2, 4, 8, 16, 32, 48, and 64 SRT samples, and the number of spots ranging from approximately 10 000 to 320 000. In this way, the simulated data are representative of current computational challenges posed by existing large-scale datasets.

Using our simulated SRT data, we found that spatialMNN is both accurate, based on ARI and normalized mutual information (NMI) (Fig. 5d and e, Fig. 8a and b, available as supplementary data at *Bioinformatics* online) and memory efficient (Fig. 5f, Fig. 8d, available as supplementary data at *Bioinformatics* online), as spatialMNN can identify spatial domains from *N *= 64 SRT samples with less than 5 GB of memory in the absence of batch effects, or approximately 11 GB even under extreme batch effects (up to 500% shift and 125% variance scaling). Furthermore, spatialMNN is computationally fast as it can identify spatial domains from *N *= 64 SRT samples in under 10 min without batch effects, and around 22 min with batch effects. Compared to existing methods, we found spatialMNN outperforms other approaches in all three categories, including accuracy, memory usage, and running time. Notably, while the accuracy performance of other methods like BASS, BANKSY, and MENDER declines as the number of samples increases, and methods like BayesSpace and SLAT show high variability in results when batch effects are introduced (Fig. 8a and b, available as supplementary data at *Bioinformatics* online), spatialMNN consistently maintains high accuracy. It demonstrates strong robustness to batch effects and scalability to large datasets. In terms of computational efficiency, spatialMNN significantly outperforms other methods, exhibiting lower memory usage and shorter runtime (Fig. 5f and g). Methods like BASS, Seurat, and MENDER display a pronounced increase in either memory consumption or computational time, and sometimes both with larger datasets, whereas spatialMNN scales more efficiently. These results highlight the ability of spatialMNN to deliver high clustering accuracy with minimal computational cost, making it a highly accurate, efficient, and scalable solution for large-scale spatial transcriptomics analysis.

## 4 Discussion

Here we introduce spatialMNN, a new approach to integrate multiple SRT data and identify spatial domains across all samples. The computing performance of spatialMNN is greatly increased compared to other algorithms due to its reduced computational complexity via divide-and-conquer strategy, as well as the straightforward implementation of parallelization in the first stage of spatialMNN. To reduce the memory burden even further, future work could focus on the use of delayed operations to utilize data stored on-disk rather than in memory. As the accessibility to SRT technologies increase, the scale of datasets will inevitably rise too. Regardless, we have shown that spatialMNN can scale well for these datasets, as shown by the method’s strong performance on one of the largest publicly available SRT datasets.

The spatialMNN approach results in spatial domains that are consistent across multiple samples. Therefore, no per-sample clustering to *post-hoc* matching of clusters across samples is needed, ultimately reducing the burden of manual examination by analysis. Furthermore, the abundance of spatial domains identified can be used to extract a unique and deeper understanding of a set of samples, as we showed with the AD dataset (Fig. 3), which could be used to identify disease-related niches. In particular, when spatialMNN is applied to point-based SRT datasets, the clusters identified are actually spatial domain types. These are regions where a certain distribution of gene expression is observed, which can arise due to distinct demographics of cell type mixtures. Hence, it is important to note that the spatial domains found in spatialMNN will not always correspond to cell type identities.

In applying spatialMNN, despite spatialMNN including several hyperparameters—such as the number of PCs, edge pruning threshold, k-nearest neighbors, and clustering resolution—given spatialMNN’s computational efficiency, users can explore parameter settings guided not only by quantitative metrics (e.g. silhouette score) but also by biological insights, such as known spatial marker patterns or tissue morphology. To support diverse datasets and experimental platforms, we provide guidance in the supplementary section and documentation to help users adapt spatialMNN to their specific biological context.

Overall, spatialMNN is a novel approach that utilizes MNN and was inspired by the need for less time-intensive and more accurate clustering methods for multi-sample SRT datasets. With an increase in the number of large-scale SRT datasets, it will be important to ensure we can perform unified analyses. spatialMNN enables unified clustering to identify spatial domains across many samples, and is implemented with popular data structures, Seurat v5 object (Hao *et al.* 2024), which is used for storing SRT datasets and clustering results.

## Supplementary Material

btaf403_Supplementary_Data
