# Supplementary material for: Spatial mutual nearest neighbors for spatial transcriptomics data
Source: Bioinformatics. 2025 Jul 26;41(8):btaf403. doi: 10.1093/bioinformatics/btaf403 (PMC12317746; doi:10.1093/bioinformatics/btaf403)
Supplement: btaf403_Supplementary_Data [file btaf403_supplementary_data.pdf]

Supplementary Materials

Spatial mutual nearest neighbors for spatial transcriptomics data

Haowen Zhou, Pratibha Panwar, Boyi Guo, Caleb Hallinan, Shila Ghazanfar*, Stephanie C. Hicks*

*Correspondence to shila.ghazanfar@sydney.edu.au and shicks19@jhu.edu

Contents

1. **Supplemental Notes 1-2**
2. **Supplementary Figures 1-8**

Supplemental Notes

Note 1: Description of publicly available datasets used

SpatialMNN can be applied to either (i) image-based, targeted, in situ transcriptomic profiling at a molecular and single-cell resolution or (ii) non-targeted RNA capture and sequencing approaches [1].

Mouse brain STARmap data

Gene expression counts were downloaded from [14] who generated on the STARmap (spatially resolved transcript amplicon readout mapping) platform profiling the prelimbic area in mouse brain across N=3 tissue sections, 166 genes, and n=3,190 cells. Cell labels were assigned *a priori* to each cell in the original publication. We note that the BayesSpace algorithm could not be run on this dataset because it is not applicable to image-based *in situ* technologies profiling at a molecular and single-cell resolution.

Visium human dorsolateral prefrontal cortex data

Gene expression counts were downloaded from Globus from [7]. These data were generated on the 10x Genomics Visium Spatial Gene Expression platform profiling postmortem dorsolateral prefrontal cortex (DLPFC) in human brain across N=12 tissue sections, 33,538 genes, and n=47,681 spots. Spatial domains were manually annotated at the spot-level *a priori* in the original publication.

MERFISH mouse frontal cortex and striatum data

Gene expression counts were downloaded from CZI CELLxGENE Collections portal, which were published in [3]. These data were generated on the MERFISH platform profiling frontal cortex and striatum in mouse brain across N=31 tissue sections, 374 genes, and n=378,918 cells. Cell labels were assigned *a priori* to each cell in the original publication. We note that the BayesSpace algorithm could not be run on this dataset because it is not applicable to image-based *in situ* technologies profiling at a molecular and single-cell resolution. Further, BASS could not be run on this dataset due to out-of-memory issue.

STARmap PLUS Alzheimer's Disease mouse brain data

Gene expression counts were downloaded from [17] who generated on the STARmap PLUS (protein localization and unlimited sequencing) platform profiling cortical and hippocampal regions in TauPS2APP triple transgenic mice, an established AD mouse model that exhibits both amyloid plaque and tau pathologies. Gene expression and protein levels were measured in N=4 tissue sections from 8- and 13-month-old TauPS2APP mouse and normal mouse brains in the context of extracellular amyloid-β (Aβ) plaques and intracellular hyperphosphorylated tau accumulation. A targeted list of 2,766 genes were profiled measuring n=35,094 cells and 2 proteins.

MERFISH mouse hypothalamic preoptic region data

Gene expression counts were downloaded from [8] who generated on the MERFISH platform profiling mouse hypothalamic preoptic regions from 6 social behaviour types in adult C57Bl6/J 16 female and 20 male mice. A targeted panel of 161 genes was measured in n=1,027,848 cells from N=181 tissue sections, and cell labels were assigned *a priori* to each cell in the original publication. After filtering out cells with "Ambiguous" label, 874,768 cells were used in the benchmark.

Visium human hippocampus data

Gene expression counts were downloaded from [9] who generated on the 10x Genomics Visium Spatial Gene Expression platform postmortem hippocampus (HPC) in human brain across N=31 tissue sections from 10 neurotypical donors (2-5 Visium slides per donor), 31,483 genes, and n=150,917 spots.

Note 2: Existing computational methods for multi-sample spatial domain detection

We compared spatialMNN to existing methods that integrate multiple SRT tissue samples and perform unsupervised clustering. In our benchmark evaluations, we used default parameters obtained from the developer-provided tutorials or publications, unless noted below.

BASS

BASS [5] performs unsupervised clustering across multiple SRT samples either at a single-cell resolution or for spatial domain detection using a Bayesian hierarchical modeling framework. It takes as input a list of raw gene expression matrices and spatial coordinates of cells or spots, along with sample information like the expected number of cell types (C) and spatial domains (R). These parameters are not optional and the user needs to provide some values for these when creating BASS object. BASS provides the outputs including the inferred cell type cluster labels, spatial domain labels, and the cell type proportions inside each spatial domain. In the benchmark, we used the spatial domain labels (z) to calculate the accuracy metrics. We used the recommended parameters in benchmark: For STARmap datasets, the region number is set to 4 and top 10 PCs are used in the preprocessing step. Region number and the number of PCs are set to 7 and 20 respectively for DLPFC dataset. The software is available on GitHub (github.com/zhengli09/BASS) and we used version 1.6.5.

PRECAST

PRECAST [6] is a probabilistic framework designed for joint analysis of multiple spatial transcriptomics datasets. It integrates spatial coordinates and gene expression profiles to identify spatial domains while accounting for batch effects across samples. PRECAST takes as input the spatial locations and gene expression matrices of multiple SRT datasets, optionally incorporating covariates or batch labels. As output, it provides spatially coherent domain clusters across datasets while simultaneously correcting for technical variations, improving the biological interpretability of spatial domain assignments. The number of cluters is set to 4 (STARmap), 7 (DLPFC), and 8 (MERFISH) respectively. The software is available on CRAN (cloud.r-project.org/web/packages/PRECAST/index.html) and we used version 1.1.0.016.

BayesSpace

BayesSpace [18] enhances spatial resolution in SRT data by refining spatial domain boundaries using a Bayesian modeling approach. It models spatial expression data through a mixture model that accounts for neighboring relationships between spots to improve the detection of fine-grained spatial domains. BayesSpace takes as input spatial gene expression data from a standard SRT experiment and optional neighborhood information. As output, it provides high-resolution spatial domain assignments, offering a refined clustering of the spatial transcriptomics data that accounts for local dependencies and spatial structure. Based on its recommended type of SRT platforms, we only run BayesSpace on the DLPFC and simulated datasets. The first 50 PCs are used for spatial preprocessing and the number of clusters is set to 7 and 4 respectively. The software is available on Bioconductor (www.bioconductor.org/packages/release/bioc/html/BayesSpace.html), and we used BayesSpace version 1.6.0.

BANKSY

BANKSY [11] performs unsupervised clustering at spatial domain or cell type level using the gene expressions of cells as well as their neighbourhood representations built using weighted average of neighbourhood gene expressions and an azimuthal Gabor filter. It takes a SpatialExperiment object [10] as input, and the cluster labels generated by the algorithm are added to the metadata in the object. We followed BANKSY preprocessing steps such as trimming the datasets to save memory. For datasets with

samples from different sources, additional preprocessing steps such as staggering spatial coordinates to avoid overlap between locations from different samples and running harmony for batch correction were performed. Whole transcriptome spatial transcriptomics datasets, like the DLPFC dataset, were subjected to feature selection using FindVariableFeatures function (nfeatures = 2000) of Seurat [12], prior to batch correction and in accordance with BANKSY protocol. The selection of BANKSY parameters such as neighbourhood size (k_geom), mixing parameter (lambda), Leiden clustering resolution (res), and number of PCA dimensions (npcs) depended on the type of input dataset. We used BANKSY recommended values for domain-level clustering – for DLPFC dataset k_geom = 18, lambda = 0.2, res = 0.55, npcs = 20; for STARmap dataset k_geom = 30, lambda = 0.8, res = 0.8, npcs = 50; and for MERFISH dataset k_geom = 30, lambda = 0.8, res = 1, npcs = 20. The software is available on GitHub (github.com/prabhakarlab/Banksy), and we used BANKSY version 1.0.0.

SLAT

SLAT [15] is a graph convolutional network-based alignment tool capable of mapping multiple samples from different technologies and modalities using a graph adversarial matching algorithm. For multisample alignment, SLAT requires a list of AnnData objects [13] arranged in the correct order for 3D reconstruction of original tissue. The output is a list of best matching cell indices from each pair of samples aligned. For benchmarking purpose, we added a Leiden clustering step prior to SLAT alignment, such that clustering was performed on the first sample in the AnnData object list, followed by multisample alignment, and then sequential borrowing of cluster labels from first to second sample, second to third sample, and so on. To ensure fair treatment, we performed this clustering + SLAT approach using each sample in the dataset as the first clustered sample. To maximize the number of alignments in SLAT, we used a low cosine similarity cutoff of 0.3. SLAT software is available on GitHub (github.com/gao-lab/SLAT), and we used SLAT version 0.2.1.

MENDER

MENDER [16] is a multisample, clustering method that performs spatial domain detection via a multi-range neighbourhood representation approach. MENDER takes AnnData objects as input, and it requires cell state information in the form of prior cell annotations or cluster labels to generate spatial context for each cell over a range of neighbourhood sizes. MENDER output contains an AnnData object with MENDER cluster labels. For benchmarking, we used Harmony algorithm [4] on samples from different sources to counter batch effects and Leiden clustering to generate cell state information, as recommended. MENDER parameters such as number of neighbourhood sizes (n_scales), neighbourhood mode based on the spatial technology of input dataset (nn_mode), neighbourhood size (nn_para), and final Leiden clustering resolution (target_k) were selected based on the type of input data. We followed MENDER tutorial for making these parameter selections for each dataset – for DLPFC dataset n_scales = 6, nn_mode = ‘ring’, nn_para = 6, and target_k = -0.2; for STARmap and MERFISH datasets n_scales = 6, nn_mode = ‘radius’, target_k = -0.5/8, and nn_para = 150/15 (STARmap/MERFISH). The software is available on GitHub (github.com/yuanzhiyuan/MENDER), and we used MENDER version 1.1.

Louvain method for community detection (Seurat)

Louvain clustering [2], which is embedded in Seurat v3 [12], is a graph-based approach for detecting clusters in single-cell data. It operates by constructing a shared Nearest-neighbor (SNN) graph and optimizing the modularity to detect communities or clusters. Louvain takes as input a precomputed KNN graph derived from an expression matrix, along with a resolution parameter that controls cluster granularity. The output is a set of clusters representing either cell types or spatial domains, with flexibility in adjusting the resolution to obtain finer or broader clusters. As recommended by Seurat tutorial, the raw count matrices of all datasets are used as input. Followed by scaling, normalization and

HVG selection, the dimension of the expression matrices are further reduced by PCA. For SNN construction, we used top 10 PCs and default resolution (0.1) for clustering. The software is available on CRAN (cran.r-project.org/web/packages/Seurat/index.html), and we used Seurat version 5.0.3.

Supplemental Figures

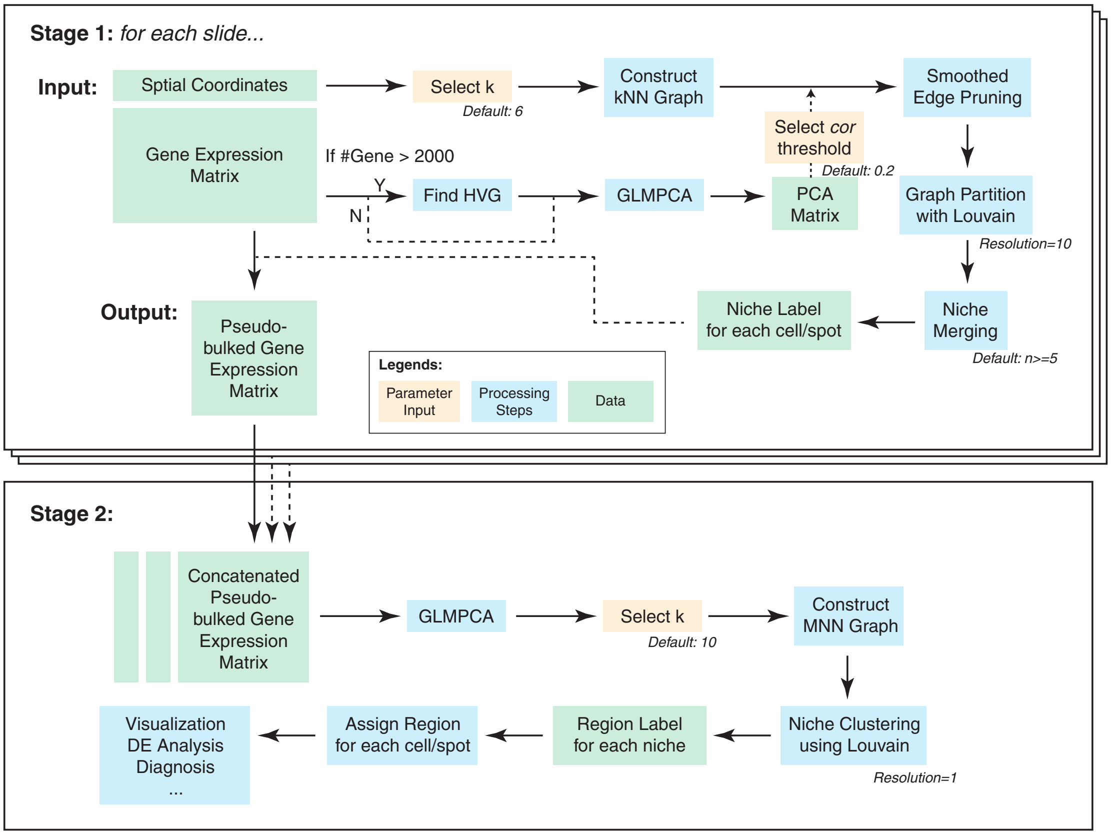

Supplementary Figure 1: The detailed schematic of the two-stage pipeline used in spatialMNN. Stage 1 begins with input data consisting of spatial coordinates and gene expression matrices for each slide. Initially, a k-nearest neighbor (kNN) graph is constructed (default $k = 6$) based on spatial distances. Highly variable genes (HVGs) are identified if the number of genes exceeds 2000. Generalized Linear Model Principal Component Analysis (GLMPCA) is applied to reduce dimensionality, producing a PCA matrix. A correlation threshold (default $cor = 0.2$) is set, and smoothed edge pruning is performed to refine the graph. The correlation threshold can be determined from the weight distribution of all edges. Typically the threshold will be higher if applying SpatialMNN on spot-based platforms. The graph is then partitioned using the Louvain algorithm with a resolution parameter of 10, and niche merging is conducted (default $n < 5$). The output of this stage is a pseudo-bulked gene expression matrix and niche labels for each cell or spot. Stage 2 involves the concatenation of pseudo-bulked gene expression matrices across slides. GLMPCA is applied again for dimensionality reduction, followed by kNN graph construction (default $k = 10$) and the construction of a mutual nearest neighbor (MNN) graph. Niche clustering is performed using the Louvain algorithm (resolution $= 1$), resulting in region labels for each niche. Downstream analysis like visualization, differential expression analysis, and diagnosis insights for each region can be done using spatialMNN output labels.

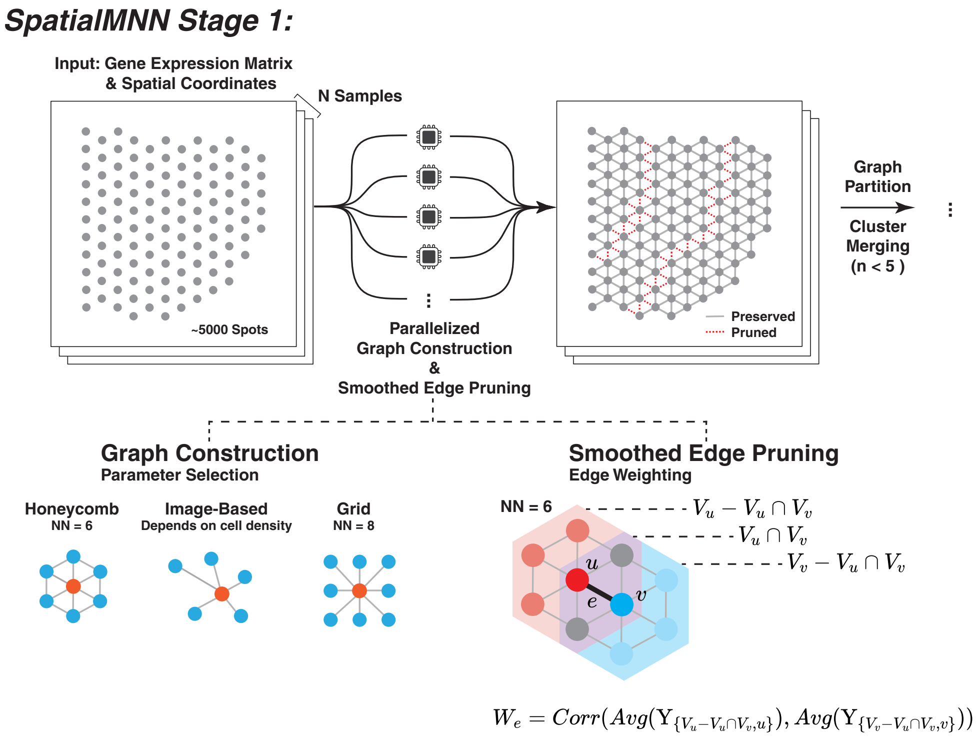

Supplementary Figure 2: Overview of the smoothed edge pruning algorithm. Parallelized graph construction and edge pruning are applied independently to each sample, with input of gene expression matrices and spatial coordinates. Various graph topologies, such as honeycomb (suitable for 10X Visium, NN = 6) and grid (NN = 8), are used to represent spatial relationships between spots. For image-based SRT technologies, such as MERFISH and STARmap, the NN is dependent on cell density. Smoothed edge pruning is then performed by calculating the correlation of average gene expression between neighboring spots, where W_e is the correlation between the gene expression of neighboring nodes after excluding their shared neighbors. This process removes weak or noisy connections, preserving only significant spatial relationships. The refined graph is subsequently partitioned and clusters are merged (typically $n < 5$), facilitating robust identification of spatial niches.

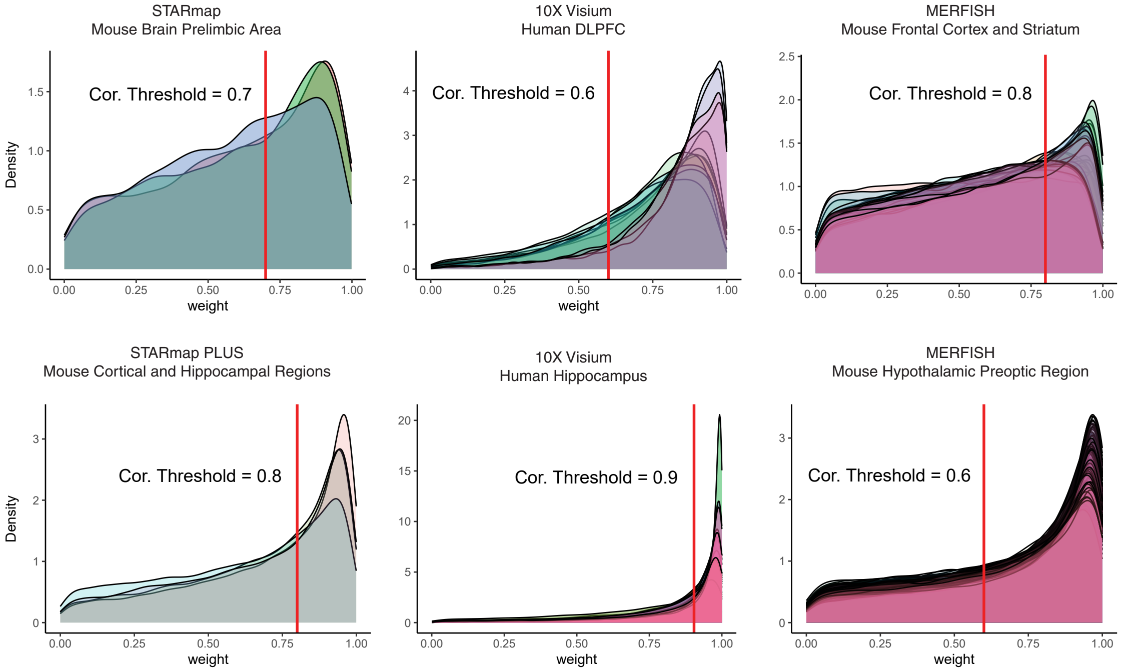

Supplementary Figure 3: Edge Weight Distribution and threshold used in spatialMNN. The weight distribution of the spatial coordinates-based graph constructed in the stage 1, the distribution of each sample is displayed in different colors, and the threshold value used for the graph pruning in stage 1 is marked with a red vertical line. The value is determined according to the elbow point of the overall weight distribution.

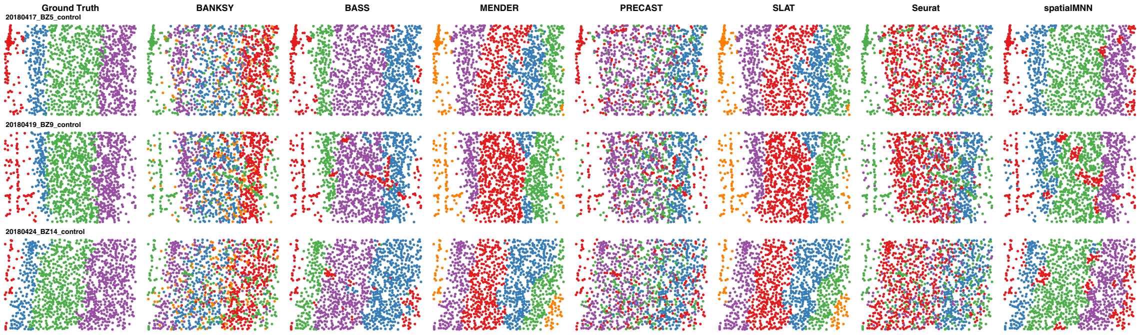

Supplementary Figure 4: Benchmark results of STARmap mouse brain prelimbic area data.

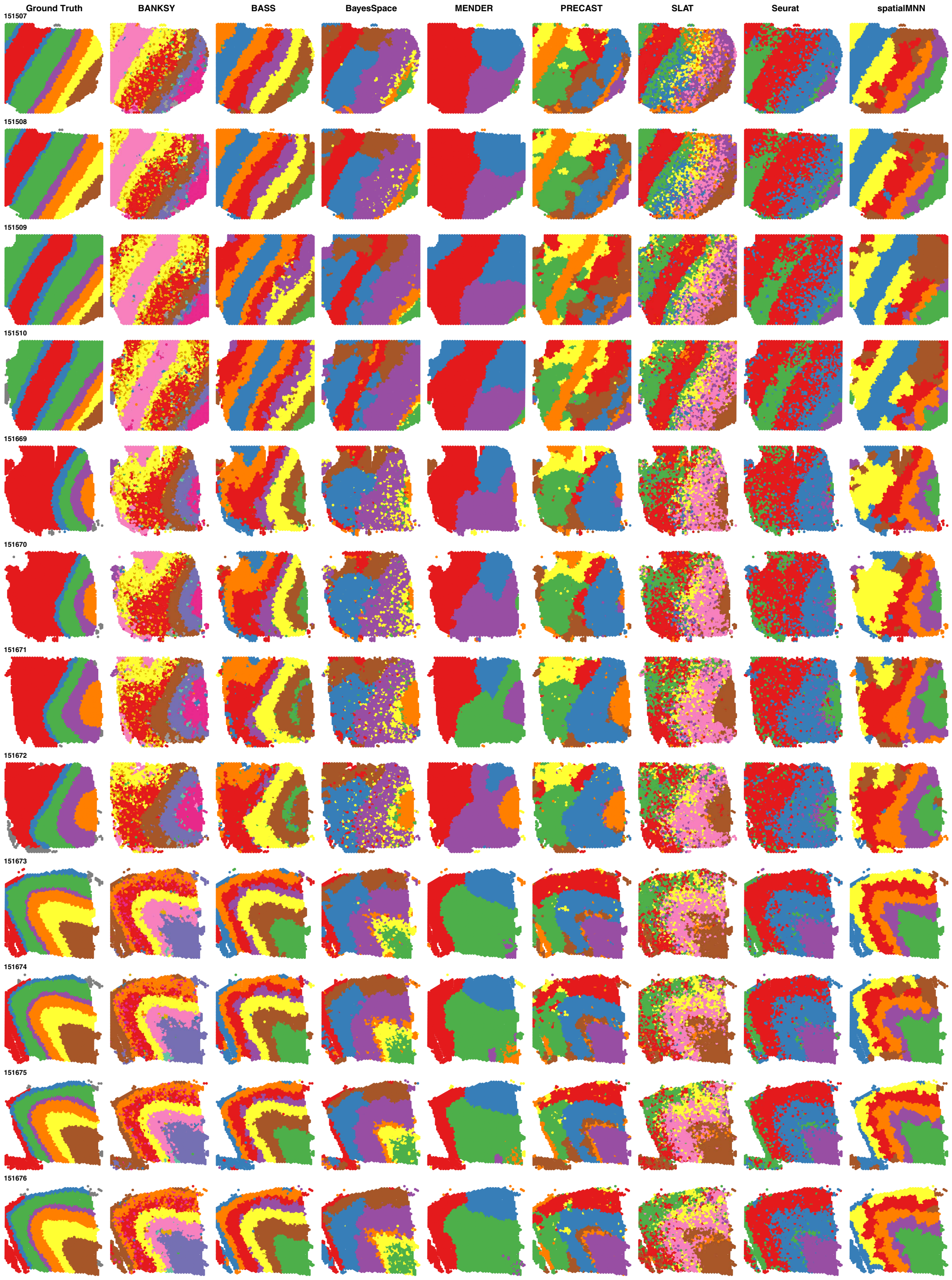

Supplementary Figure 5: **Benchmark results of Visium human dorsolateral prefrontal cortex data**.

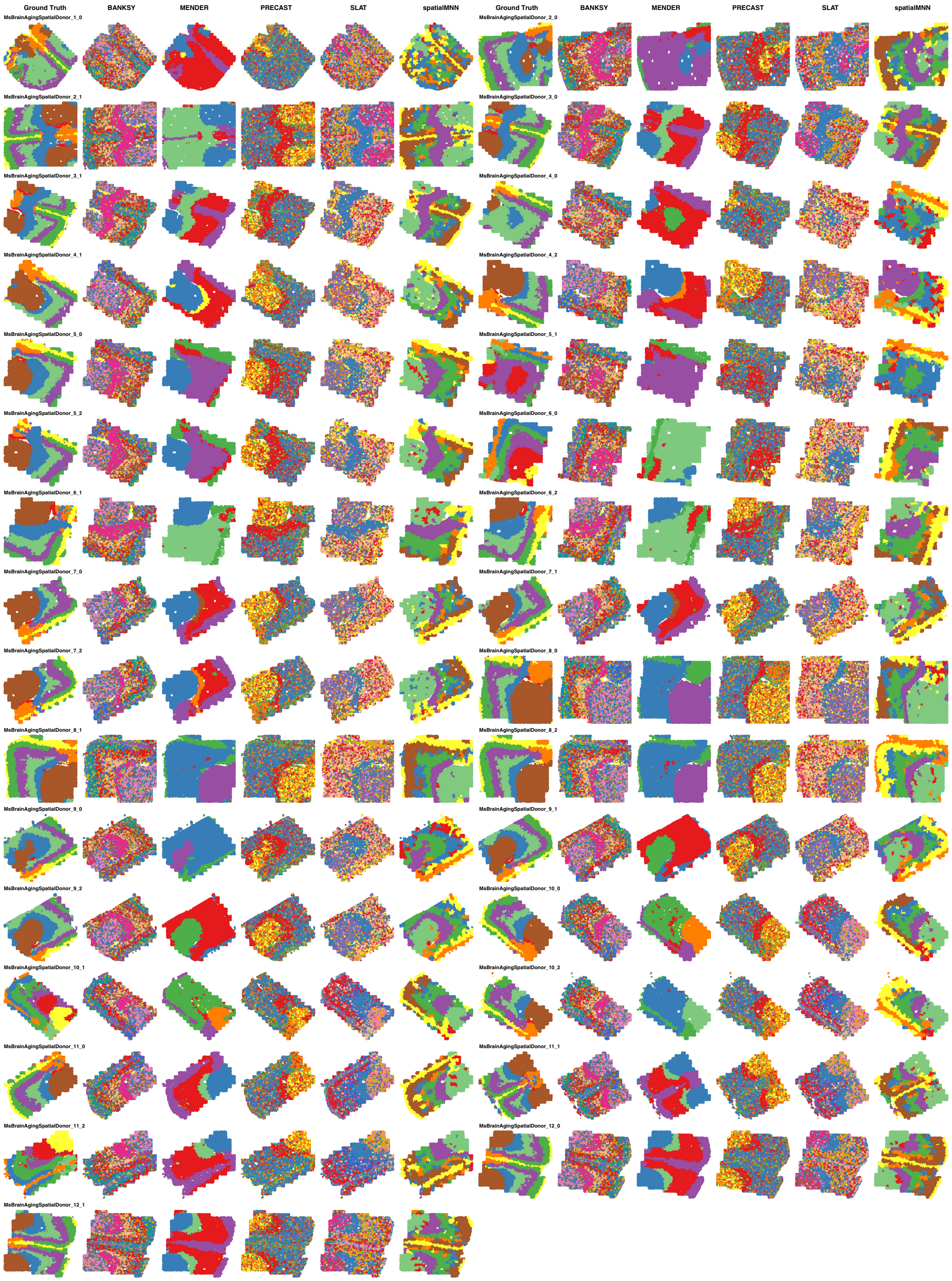

Supplementary Figure 6: **Benchmark results of MERFISH mouse frontal cortex and striatum data**.

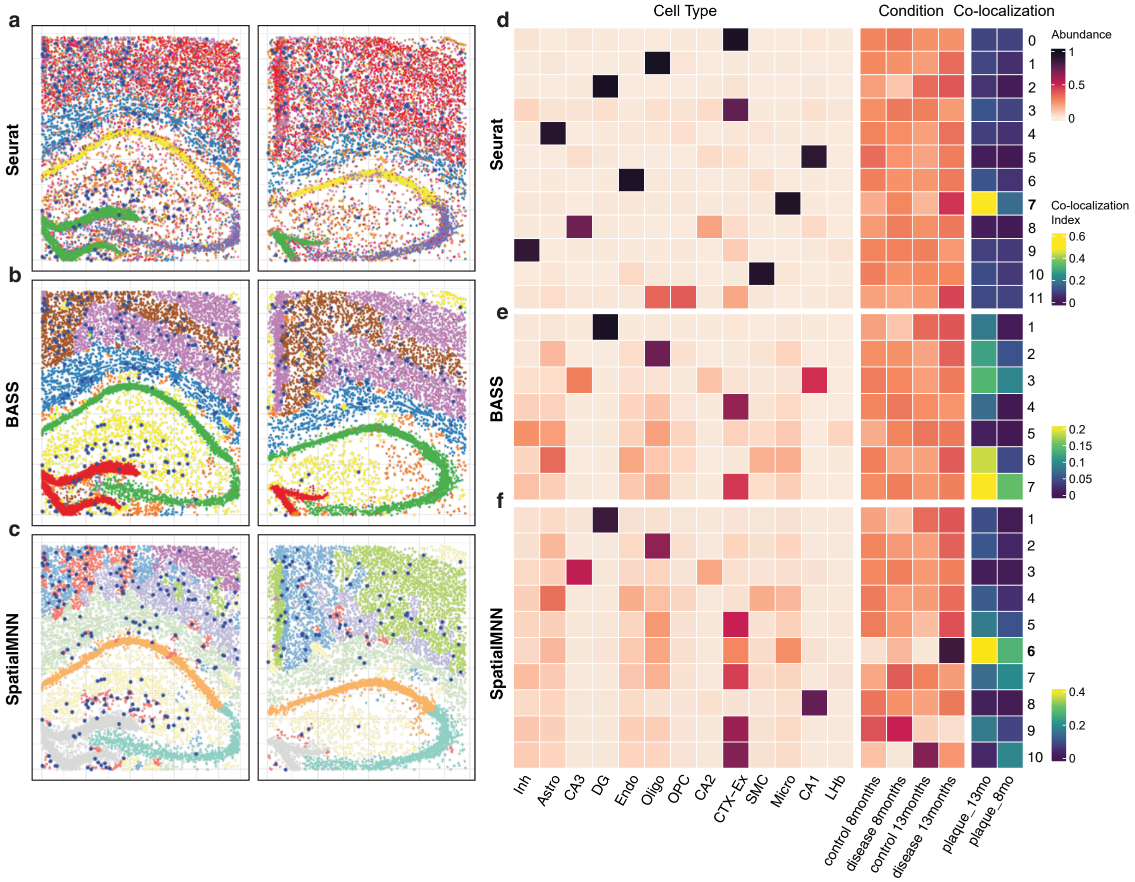

Supplementary Figure 7: Comparison of Seurat, BASS and spatialMNN running results on STARmap AD datasets. (a–c) Visualization of clustering results for the 13-month (left) and 8-month (right) AD samples using Seurat (a), BASS (b), and spatialMNN (c). (d–f) Heatmaps showing cell type abundance (left), sample condition abundance (middle), and co-localization index with Aβ plaques (right) for Seurat (d), BASS (e), and spatialMNN (f). Seurat successfully identified major cell types such as microglia and astrocytes, but failed to resolve the complex multicellular structures around plaques. BASS detected a plaque-associated cluster (Cluster 7), but with weak spatial correlation and no enrichment in AD samples. In contrast, spatialMNN uniquely captured both the spatial organization and biological relevance of disease-associated niches, highlighting its advantage in spatial domain detection in AD pathology.

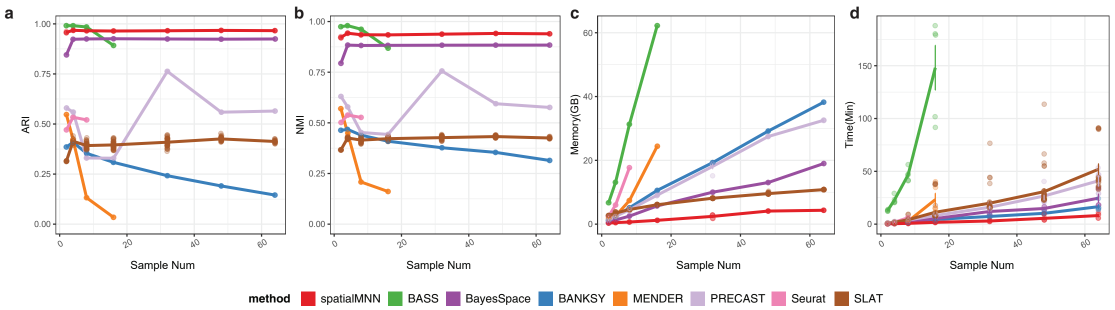

Supplementary Figure 8: Benchmark results of simulated dataset without batch effects. (a-d) Benchmarking results comparing spatialMNN with other methods (BASS, Banksy, BayesSpace, MENDER, PRECAST, Seurat, and SLAT) on **(d)** Adjusted Rand Index (ARI), **(e)** Normalized Mutual Information (NMI), **(f)** memory usage, and **(g)** runtime across different sample sizes. Each simulated benchmark with different sample size was repeated 5 times. SpatialMNN demonstrates high clustering accuracy with consistently high ARI and NMI scores, while maintaining significantly lower computational costs in terms of runtime and memory usage.

References

[1] Lyla Atta and Jean Fan. Computational challenges and opportunities in spatially resolved transcriptomic data analysis. *Nat Commun*, 12(1):5283, September 2021.

[2] Vincent D Blondel, Jean-Loup Guillaume, Renaud Lambiotte, and Etienne Lefebvre. Fast unfolding of communities in large networks. *J. Stat. Mech.*, 2008(10):P10008, October 2008.

[3] Julia Joung, Sai Ma, Tristan Tay, Kathryn R. Geiger-Schuller, Paul C. Kirchgatterer, Vanessa K. Verdine, Baolin Guo, Mario A. Arias-Garcia, William E. Allen, Ankita Singh, Olena Kuksenko, Omar O. Abudayyeh, Jonathan S. Gootenberg, Zhanyan Fu, Rhiannon K. Macrae, Jason D. Buenrostro, Aviv Regev, and Feng Zhang. A transcription factor atlas of directed differentiation. *Cell*, 186(1):209–229.e26, January 2023.

[4] Ilya Korsunsky, Jean Fan, Kamil Slowikowski, Fan Zhang, Kevin Wei, Yuriy Baglaenko, Michael Brenner, Po-Ru Loh, and Soumya Raychaudhuri. Fast, sensitive, and accurate integration of single cell data with harmony. *bioRxiv*, 2018.

[5] Zheng Li and Xiang Zhou. BASS: multi-scale and multi-sample analysis enables accurate cell type clustering and spatial domain detection in spatial transcriptomic studies. *Genome Biology*, 23(1):168, August 2022.

[6] Wei Liu, Xu Liao, Ziye Luo, Yi Yang, Mai Chan Lau, Yuling Jiao, Xingjie Shi, Weiwei Zhai, Hongkai Ji, Joe Yeong, and Jin Liu. Probabilistic embedding, clustering, and alignment for integrating spatial transcriptomics data with PRECAST. *Nature Communications*, 14(1):296, January 2023.

[7] Kristen R Maynard, Leonardo Collado-Torres, Lukas M Weber, Cedric Uytingco, Brianna K Barry, Stephen R Williams, Joseph L Catallini, 2nd, Matthew N Tran, Zachary Besich, Madhavi Tippani, Jennifer Chew, Yifeng Yin, Joel E Kleinman, Thomas M Hyde, Nikhil Rao, Stephanie C Hicks, Keri Martinowich, and Andrew E Jaffe. Transcriptome-scale spatial gene expression in the human dorsolateral prefrontal cortex. *Nat Neurosci*, 24(3):425–436, 2021.

[8] Jeffrey R Moffitt, Dhananjay Bambah-Mukku, Stephen W Eichhorn, Eric Vaughn, Karthik Shekhar, Julio D Perez, Nimrod D Rubinstein, Junjie Hao, Aviv Regev, Catherine Dulac, and Xiaowei Zhuang. Data from: Molecular, spatial and functional single-cell profiling of the hypothalamic preoptic region, 2018.

[9] Erik D. Nelson, Madhavi Tippani, Anthony D. Ramnauth, Heena R. Divecha, Ryan A. Miller, Nicholas J. Eagles, Elizabeth A. Pattie, Sang Ho Kwon, Svitlana V. Bach, Uma M. Kaipa, Jianing Yao, Joel E. Kleinman, Leonardo Collado-Torres, Shizhong Han, Kristen R. Maynard, Thomas M. Hyde, Keri Martinowich, Stephanie C. Page, and Stephanie C. Hicks. An integrated single-nucleus and spatial transcriptomics atlas reveals the molecular landscape of the human hippocampus. *bioRxiv: The Preprint Server for Biology*, page 2024.04.26.590643, April 2024.

[10] Dario Righelli, Lukas M Weber, Helena L Crowell, Brenda Pardo, Leonardo Collado-Torres, Shila Ghazanfar, Aaron T L Lun, Stephanie C Hicks, and Davide Risso. SpatialExperiment: infrastructure for spatially-resolved transcriptomics data in R using bioconductor. *Bioinformatics*, 38(11):3128–3131, May 2022.

[11] Vipul Singhal, Nigel Chou, Joseph Lee, Yifei Yue, Jinyue Liu, Wan Kee Chock, Li Lin, Yun-Ching Chang, Erica Mei Ling Teo, Jonathan Aow, Hwee Kuan Lee, Kok Hao Chen, and Shyam Prabhakar. BANKSY unifies cell typing and tissue domain segmentation for scalable spatial omics data analysis. *Nat. Genet.*, 56(3):431–441, March 2024.

[12] Tim Stuart, Andrew Butler, Paul Hoffman, Christoph Hafemeister, Efthymia Papalexi, William M Mauck, 3rd, Yuhan Hao, Marlon Stoeckius, Peter Smibert, and Rahul Satija. Comprehensive integration of single-cell data. *Cell*, 177(7):1888–1902.e21, June 2019.

[13] Isaac Virshup, Sergei Rybakov, Fabian J Theis, Philipp Angerer, and F Alexander Wolf. anndata: Annotated data. *bioRxiv*, December 2021.

[14] Xiao Wang, William E. Allen, Matthew A. Wright, Emily L. Sylwestrak, Nikolay Samusik, Sam Vesuna, Kathryn Evans, Cindy Liu, Charu Ramakrishnan, Jia Liu, Garry P. Nolan, Felice-Alessio Bava, and Karl Deisseroth. Three-dimensional intact-tissue sequencing of single-cell transcriptional states. *Science (New York, N.Y.)*, 361(6400):eaat5691, July 2018.

[15] Chen-Rui Xia, Zhi-Jie Cao, Xin-Ming Tu, and Ge Gao. Spatial-linked alignment tool (SLAT) for aligning heterogenous slices. *Nat. Commun.*, 14(1):1–12, November 2023.

[16] Zhiyuan Yuan. MENDER: fast and scalable tissue structure identification in spatial omics data. *Nat. Commun.*, 15(1):1–17, January 2024.

[17] Hu Zeng, Jiahao Huang, Haowen Zhou, William J Meilandt, Borislav Dejanovic, Yiming Zhou, Christopher J Bohlen, Seung-Hye Lee, Jingyi Ren, Albert Liu, Zefang Tang, Hao Sheng, Jia Liu, Morgan Sheng, and Xiao Wang. Integrative in situ mapping of single-cell transcriptional states and tissue histopathology in a mouse model of alzheimer's disease. *Nat. Neurosci.*, 26(3):430–446, March 2023.

[18] Edward Zhao, Matthew R Stone, Xing Ren, Jamie Guenthoer, Kimberly S Smythe, Thomas Pulliam, Stephen R Williams, Cedric R Uytingco, Sarah E B Taylor, Paul Nghiem, Jason H Bielas, and Raphael Gottardo. Spatial transcriptomics at subspot resolution with BayesSpace. *Nat Biotechnol*, 39(11):1375–1384, November 2021.
